# Human Papillomavirus vaccine coverage among female students in Brunei Darussalam: results from the first 4 years of the national school-based vaccination programme

**DOI:** 10.1016/j.heliyon.2019.e02588

**Published:** 2019-10-18

**Authors:** Li Ling Chaw, Shaun Tze Wei Lim, Siti Rosemawati Md Yussof

**Affiliations:** a PAPRSB Institute of Health Sciences, Universiti Brunei Darussalam, Brunei; b Ministry of Health, Brunei

**Keywords:** Health sciences, Public health, Infectious disease, Women's health, Social sciences, Information science, Human papillomavirus, HPV, Vaccination, Females, Students, Brunei

## Abstract

**Background:**

Human Papillomavirus (HPV) is the leading cause of cervical cancer. HPV vaccination among girls (ages 9–14) is an effective way to prevent infection. Since 2012, Brunei Darussalam has implemented the National School-based HPV Vaccination programme which aims to vaccinate all female school students of 10–17 years old. This study was conducted to calculate and descriptively compare the coverage rates of all private and government secondary school students at both national and district levels, along with the parental consent rate.

**Methods:**

HPV vaccination records of all female students between January 2012 and December 2015 were retrospectively extracted from the School Health Services, Ministry of Health. Descriptive statistics were used to report the overall and annual vaccination coverage rate (by district, class year, nationality and type of school) and parental consent rate.

**Results:**

A total of 27,178 female students were recorded during the study period, with an overall complete dose coverage rate of 85.8% (95% CI: 85.4%, 86.2%) and 90.8% (95% CI: 90.4%, 91.2%) for all and Bruneian female students, respectively. A similar trend could be observed each year, where there is a decrease in the coverage rate from the first, second and complete doses. Brunei-Muara had the lowest vaccination coverage and parental consent rates among the country's four districts. We also observed higher HPV vaccination coverage rate for government students. Parental consent rate of Bruneian students were considerably higher than that of non-Bruneian students.

**Conclusion:**

Overall, the national school-based vaccination programme has achieved a high complete dose coverage rate in its first 4 years of implementation. Issues identified for this programme are vaccine cost and difficulty to reach students who have missed their scheduled vaccination in schools. The programme can be further improved by identifying other barriers of accepting and completing their HPV vaccine dose schedule.

## 1 Introduction

Human Papillomavirus (HPV) infection is the leading cause of cervical cancer, which is the fourth most common cancer in women globally [1]. The World Health Organisation (WHO) estimated that in 2018 there were 570,000 new cases of cervical cancer, and approximately 311,000 fatalities from the disease [1].

Cervical cancer can be prevented through HPV vaccination. Such vaccines mainly protect against HPV subtypes 16 and 18 infection, which have been known to cause 70% of cervical cancers worldwide [2]. Previous population-based studies have reported significantly lower rates of cervical abnormalities among HPV-vaccinated women, when compared to those unvaccinated [3, 4]. A systematic review has reported that countries with a female HPV vaccination coverage of at least 50% have significantly decreased levels of HPV prevalence, genital warts, and cervical intraepithelial neoplasia [5]. The WHO recommends giving HPV vaccination to girls of between 9 - 14 years old, before their sexual debut as the vaccine is most effective if given prior to exposure [1].

Since its introduction in 2006, many countries have employed different types of HPV vaccination programmes, either at the national or provincial level [6]. The coverage rates between these countries varied considerably in terms of programme design and performance; ranging from >80% in countries such as Scotland [7], Canada [8] and Australia [9] to <50% in France [10] and Japan [11].

Several factors have been reported as possible barriers of HPV vaccine uptake at the individual level: vaccine cost, lack of information, physician recommendation, parental concern on vaccine safety and also concerns that the vaccine indirectly encourages children to engage in sexual activity [12]. At the national level, other factors that would affect coverage rate include the choice of vaccine delivery strategy, engagement with key community members, and perceived health concerns caused by media coverage [13, 14]. School-based delivery of these programmes has been shown to have high vaccination coverage when compared to non-school-based approaches [15, 16].

Cervical cancer has been one of the top leading causes of cancer deaths amongst Bruneian women, and it ranked 4^th^ in 2017 in the country [17]. The Ministry of Health in Brunei Darussalam has embarked on the National School-based HPV Vaccination programme since 2012. It is one of the initiatives under the National Cervical Cancer Prevention and Control Programme, which aims to vaccinate all female students of ages 10–17 years, from both government and private schools nationwide. The target group of this programme is female students in Year 7 (ages 10–12 years), while a catch-up programme was also carried out for those in Year 11 (ages 15–17 years). In schools where the student population was small, catch-up was also conducted for female students from Years 8–10.

Since the implementation of this programme, national estimates of overall HPV vaccination rates were reportedly high. This study aims to report the results from this programme by determining and descriptively comparing the trends of HPV vaccination coverage rates at both national and district levels, in the first 4 years of its implementation (from 2012 to 2015). A secondary objective is to identify any issues that may have prevented vaccination rates from improving. Results from this study can help inform stakeholders on how successful the implementation of the national school-based HPV vaccination programme was in Brunei Darussalam and to propose recommendations on how to further improve it.

## 2 Methods

### 2.1 The Brunei Darussalam national school-based HPV vaccination programme

In Brunei Darussalam, all childhood vaccines listed under the Infectious Disease Act are scheduled to be administered when the child is between 0 and 5 years old. The HPV vaccine is not included in the list of diseases against which a child is to be vaccinated. Hence, the HPV vaccine is the only vaccine to be administered in the school settings. It is also not compulsory for females to get the HPV vaccine.

Under the national school-based HPV vaccination programme, the HPV vaccine is given on a voluntary basis where written consent from parents or guardians is required before the students can be vaccinated. The vaccine is given free of charge only for Bruneian students. Once consent is given, each student is given the complete course of three vaccine doses, whereby the second dose was is given one month after the first dose and the third dose was is given six months after the first dose. This 3-dose schedule was conducted from the year 2012–2015. From 2016 onwards, the 2-dose schedule was adopted following WHO recommendations, as studies suggest that both dose schedules result in comparable vaccine efficacy for adolescents of ages 9–14 years [18].

Every January (when school term starts), consent forms and information leaflets on the programme were distributed to the eligible female students and their parents or guardians via the schools. Prior to this, health talks were conducted for teachers and information booklets on the vaccine and programme were also distributed to each school. Consent forms that were not returned were deemed as refusal to get the vaccine. Students who consented to get the vaccines were vaccinated by health personnel from the School Health Services stationed at each district in the country. There are four districts in the country, namely Brunei-Muara, Tutong, Belait and Temburong. Out of this, Brunei-Muara is the most populated district where an average of 71.6% of the population resided during the study period [17]. Each district has one team of nurses, except Brunei-Muara which has four teams due to higher number of schools in the district.

The School Health Services unit received information of these eligible female students from the class enrolment list from each school, which in turn were entered into the national HPV registry. This registry is periodically updated with student's information, their consent status and dates of vaccination. On the designated vaccination day, a team of school health nurses would travel to the respective school to conduct the vaccinations for the first, second and third doses. To ensure timely vaccination, teachers were asked to remind the students about their vaccination schedule. A mobile text message was also sent to consented parents/guardians as a reminder for the scheduled vaccination. Each vaccination dosage given to a student will be recorded in the HPV vaccination registry form for each class. The total number of students consented and vaccinated were then calculated and summarised according to their class year. Students who were absent on the day of vaccination were scheduled another appointment to get their vaccine. In the event that there were a lot of absentees in a particular school, the school health nurses would visit the school again to administer the vaccines. For non-Bruneian students, they were asked to first visit the vaccination centre to pay for the vaccines prior to the designated vaccination day at their school. The receipt for this payment has to be brought to school on the designated vaccination day as proof of payment before they can be administered with the vaccine.

To increase public awareness on the benefits of the HPV vaccine, promotional activities (such as television and radio interviews, distributing leaflets and posters, and newspaper coverage) were conducted at the beginning of each year. Each school was also offered visits by School Health Services to deliver talks to parents and guardians on the vaccine/programme. In subsequent years, such promotional activities was expanded to include billboard signs and radio advertisements. These promotional activities were conducted by physicians from School Health Services.

### 2.2 Study design and data analysis

This is a retrospective study where available data on all female students studying in Years 7–11, in both government and private schools in the country, were collected between January 2012 and December 2015. This available data consisted of HPV vaccination records that were compiled into a tabulated format by the school health nurses. For each school, the following data were collected: class year (Year 7 to Year 11), total number of Bruneian and non-Bruneian female students, and total number of Bruneian and non-Bruneian female students who consented to the vaccines, who received the first, second and complete dose, respectively. Bruneian students are defined as those who are either citizens or permanent residents of Brunei Darussalam. Any data that were incomplete or miscalculated were individually reviewed and cross-checked with relevant staff from the School Health Services.

In this study, the female student population were divided into 2 groups: target (Year 7 students) and catch-up (Year 8–11 students). The catch-up group also included those who may have missed or refused to get the vaccine in previous years, but later decided to get the vaccine in that particular year.

The annual vaccination coverage rate was calculated by dividing the total number of female students who have received a particular dose by the total number of female student population for the particular year. A complete dose is defined as those who had received all three HPV doses during the study period. The parental consent rate was obtained by dividing the total number of female students who have given consent by the total number of female student population for the particular year. Differences in the coverage rates between years were compared using one-way ANOVA test and 95% Confidence Interval for one-sample proportion were also estimated. All analysis were all conducted using R ver. 3.6.0 [19] and Microsoft Excel.

The study was approved by Institute of Health Science Research Ethics Committee, PAPRSB Institute of Health Sciences, Universiti Brunei Darussalam (Reference number: UBD/IHS/B3/8).

## 3 Results

Between January 2012 and December 2015, a total of 27,178 eligible female students from 49 secondary schools were included in this study. Table 1 shows their demographic characteristics. The highest proportion of students were from Years 7 (51.4%) and 11 (43.0%). The majority of the female students were Bruneians (93.6%), resided in Brunei-Muara district (73.5%), and have studied in government schools (87.4%). There were no significant differences in the demographic characteristics between study years (Table 1).

**Table 1:** Demographic characteristics of female student population.

Number of female students		Year of Vaccination										p-value
		2012–2015		2012		2013		2014		2015		
		n	% (95% CI)	n	% (95% CI)	n	% (95% CI)	n	% (95% CI)	n	% (95% CI)	
Overall		27178	100	7907	29.1 (28.6,29.6)	6851	25.2 (24.7,25.7)	6350	23.4 (22.9,23.9)	6070	22.3 (21.8,22.8)	0.759
Specific Class Year	Year 7	13971	51.4 (50.8,52.0)	3752	47.5 (46.3,48.6)	3548	51.8 (50.6,53.0)	3402	53.6 (52.3,54.8)	3269	53.9 (52.6,55.1)	0.801
	Year 8	145	0.5 (0.45,0.63)	140	1.8 (1.5,2.1)	2	0.03	0	0.0	3	0.05	0.182
	Year 9	149	0.5 (0.47,0.65)	93	1.2 (1.0,1.4)	56	0.8 (0.6,1.1)	0	0.0	0	0.0	0.778
	Year 10	1226	4.5 (4.3,4.8)	510	6.4 (5.9,7.0)	319	4.7 (4.2,5.2)	391	6.2 (5.6,6.8)	6	0.1 (0.04,0.23)	0.610
	Year 11	11687	43.0 (42.4,43.6)	3412	43.2 (42.1,44.3)	2926	42.7 (41.5,43.9)	2557	40.3 (39.1,41.5)	2792	46.0 (44.7,47.3)	0.945
Grouped Class Year	Year 7	13971	51.4 (50.8,52.0)	3752	47.5 (46.3,48.6)	3548	51.8 (50.6,53.0)	3402	53.6 (52.3,54.8)	3269	53.9 (52.6,55.1)	0.801
	Year 8–11	13207	48.6 (48.0,49.2)	4155	52.5 (51.4,53.7)	3303	48.2 (47.0,49.4)	2948	46.4 (45.2,47.7)	2801	46.1 (44.9,47.4)	0.295
District	Brunei-Muara	19974	73.5 (73.0,74.0)	5811	73.5 (72.5,74.5)	5041	73.6 (72.5,74.6)	4707	74.1 (73.0,75.2)	4415	72.7 (71.6,73.8)	0.769
	Tutong	2699	9.9 (9.6,10.3)	783	9.9 (9.3,10.6)	676	9.9 (9.2,10.6)	622	9.8 (9.1,10.6)	618	10.2 (9.4,11.0)	0.824
	Belait	3773	13.9 (13.5,14.3)	1046	13.2 (12.5,14.0)	966	14.1 (13.3,15.0)	879	13.8 (13.0,14.7)	882	14.5 (13.7,15.4)	0.742
	Temburong	732	2.7 (2.5,2.9)	267	3.4 (3.0,3.8)	168	2.5 (2.1,2.9)	142	2.2 (1.9,2.6)	155	2.6 (2.2,3.0)	0.983
Status of students	Bruneian	25436	93.6 (93.3,93.9)	7410	93.7 (93.2,94.2)	6376	93.1 (92.4,93.7)	5974	94.1 (93.5,94.6)	5676	93.5 (92.9,94.1)	0.774
	Non- Bruneian	1742	6.4 (6.1,6.7)	497	6.3 (5.8,6.8)	475	6.9 (6.3,7.6)	376	5.9 (5.4,6.5)	394	6.5 (5.9,7.1)	0.959
Type of school	Government	23757	87.4 (87.0,87.8)	6779	85.7 (84.9,86.5)	5976	87.2 (86.4,88.0)	5633	88.7 (87.9,89.5)	5369	88.5 (87.6,89.2)	0.829
	Private	3421	12.6 (12.2,13.0)	1128	14.3 (13.5,15.1)	875	12.8 (12.0,13.6)	717	11.3 (10.5,12.1)	701	11.5 (10.8,12.4)	0.976

We observed a high vaccination coverage rate in the first year of the vaccination programme, with a complete dose coverage of 85.2% (95% Confidence Interval [95% CI]: 84.4%, 86.0%) for all female students in 2012 (Table 2a). Since then, the coverage rate increased steadily, reaching the highest complete dose rate of 87.2% (96% CI: 86.4%, 88.1%) in 2015. A similar trend was also observed for the Bruneian female students (Table 2b), and in both target and catch-up population (Table 3). When compared to all the female student population, the coverage rate for the Bruneian female students are higher (Table 2b), where their complete dose coverage ranges between 90.5% and 91.9%. The overall complete dose coverage rate was 85.8% (95% CI: 85.4%, 86.2%) and 90.8% (95% CI: 90.4%, 91.2%) for all and Bruneian female students, respectively. There were no significant differences in the coverage rates between study years (Table 2).

**Table 2:** School-based HPV vaccination coverage by doses received for (a) all and (b) Bruneian female students only. The percentage was calculated such that the denominator for (a) is the total number of female students, and for (b) is the total number of Bruneian female students for the indicated year(s).

(a) All female students	Year of Vaccination										p-value
	2012–2015		2012		2013		2014		2015		
	n	% (95% CI)	n	% (95% CI)	n	% (95% CI)	n	% (95% CI)	n	% (95% CI)	
First dose	24213	89.1 (88.7,89.5)	6900	87.3 (86.5,88.0)	6086	88.8 (88.1,89.6)	5714	90.0 (89.2,90.7)	5513	90.8 (90.1,91.5)	0.617
Second dose	24084	88.6 (88.2,89.0)	6866	86.8 (86.1,87.6)	6047	88.3 (87.5,89.0)	5677	89.4 (88.6,90.1)	5494	90.5 (89.7,91.2)	0.610
Complete dose	23318	85.8 (85.4,86.2)	6737	85.2 (84.4,86.0)	5787	84.5 (83.6,85.3)	5499	86.6 (85.7,87.4)	5295	87.2 (86.4,88.1)	0.638
(b) Bruneian female students only	2012–2015		2012		2013		2014		2015		p-value
	n	% (95% CI)	n	% (95% CI)	n	% (95% CI)	n	% (95% CI)	n	% (95% CI)	
First dose	23974	94.3 (94.0,94.5)	6860	92.6 (92.0,93.2)	6016	94.4 (93.8,94.9)	5664	94.8 (94.2,95.4)	5434	95.7 (95.2,96.2)	0.654
Second dose	23851	93.8 (93.5,94.1)	6829	92.2 (91.5,92.8)	5978	93.8 (93.1,94.3)	5629	94.2 (93.6,94.8)	5415	95.4 (94.8,95.9)	0.649
Complete dose	23096	90.8 (90.4,91.2)	6703	90.5 (89.8,91.1)	5721	89.7 (88.9,90.5)	5453	91.3 (90.5,92.0)	5219	91.9 (91.2,92.6)	0.670

**Table 3:** School-based HPV vaccination coverage by doses received for (a) Year 7 female students (Target population) and (b) Years 8–11 female students (Catch-up population). The percentage was calculated such that the denominator for (a) is the total number of Year 7 students, and for (b) is the total number of Years 8–11 students for the indicated year(s).

(a) Year 7 female students	Year of Vaccination										p-value
	2012–2015		2012		2013		2014		2015		
	n	% (95% CI)	n	% (95% CI)	n	% (95% CI)	n	% (95% CI)	n	% (95% CI)	
First dose	12457	89.2 (88.6,89.7)	3274	87.3 (86.1,88.3)	3178	89.6 (88.5,90.5)	3046	89.5 (88.4,90.5)	2959	90.5 (89.4,91.5)	0.910
Second dose	12395	88.7 (88.2,89.2)	3257	86.8 (85.7,87.9)	3163	89.1 (88.1,90.1)	3025	88.9 (87.8,89.9)	2950	90.2 (89.2,91.2)	0.911
Complete dose	12006	85.9 (85.3,86.5)	3206	85.4 (84.3,86.6)	3050	86.0 (84.8,87.1)	2915	85.7 (84.5,86.8)	2835	86.7 (85.5,87.9)	0.864
(b) Years 8-11 female students	2012–2015		2012		2013		2014		2015		p-value
	n	% (95% CI)	n	% (95% CI)	n	% (95% CI)	n	% (95% CI)	n	% (95% CI)	
First dose	11756	89.0 (88.5,89.5)	3626	87.3 (86.2,88.3)	2908	88.0 (86.9,89.1)	2668	90.5 (89.4,91.5)	2554	91.2 (90.1,92.2)	0.218
Second dose	11689	88.5 (87.9,89.0)	3609	86.9 (85.8,87.9)	2884	87.3 (86.1,88.4)	2652	90.0 (88.8,91.0)	2544	90.8 (89.7,91.9)	0.214
Complete dose	11312	85.7 (85.0,86.2)	3531	85.0 (83.9,86.0)	2737	82.9 (81.5,84.1)	2584	87.7 (86.4,88.8)	2460	87.8 (86.5,89.0)	0.201

When compared to the other districts in Brunei Darussalam, Brunei-Muara had the lowest overall complete dose coverage rate (83.7% [95% CI: 83.1%, 84.2%]; Table 4) and also the lowest overall parental consent rate (87.7% [95% CI: 87.2%, 88.1%]; Table 5). The overall complete dose coverage rate was consistently much higher for female students who studied in government schools (89.4% [95% CI: 89.0%, 89.7%]). Lastly, the overall parental consent rate (Table 5) was considerably much higher for Bruneian students (94.4% [95% CI: 94.1%, 94.7%]) when compared with that of non-Bruneian students (14.0% [95% CI: 12.4%, 15.7%]). There were also no significant differences in the parental consent rates between study years (Table 5).

**Table 4:** School-based HPV vaccination coverage rates for complete doses, stratified by district and by type of school.

Vaccination coverage rate (for complete dose only)	Year of Vaccination										
	2012–2015		2012		2013		2014		2015		p-value
	n	% (95% CI)	n	% (95% CI)	n	% (95% CI)	n	% (95% CI)	n	% (95% CI)	
Overall	23318	85.8 (85.4,86.2)	6737	85.2 (84.4,86.0)	5787	84.5 (83.6,85.3)	5499	86.6 (85.7,87.4)	5295	87.2 (86.4,88.1)	0.638
**By district**											
Brunei-Muara	16712	83.7 (83.1,84.2)	4721	81.2 (80.2,82.2)	4171	82.7 (81.7,83.8)	4018	85.4 (84.3,86.4)	3802	86.1 (85.1,87.1)	0.581
Tutong	2527	93.6 (92.6,94.5)	770	98.3 (97.1,99.1)	611	90.4 (87.8,92.5)	572	92.0 (89.5,93.9)	574	92.9 (90.5,94.7)	0.670
Belait	3396	90.0 (89.0,90.9)	1005	96.1 (94.7,97.1)	849	87.9 (85.6,89.8)	775	88.2 (85.8,90.2)	767	87.0 (84.5,89.1)	0.800
Temburong	683	93.3 (91.2,95.0)	241	90.3 (85.9,93.4)	156	92.9 (87.6,96.1)	134	94.4 (88.8,97.4)	152	98.1 (94.0,99.5)	0.980
**By type of school**											
Government	21228	89.4 (89.0,89.7)	6071	89.6 (88.8,90.3)	5261	88.0 (87.2,88.8)	5051	89.7 (88.8,90.4)	4845	90.2 (89.4,91.0)	0.738
Private	2090	61.1 (59.4,62.7)	666	59.0 (56.1,61.9)	526	60.1 (56.8,63.4)	448	62.5 (58.8,66.0)	450	64.2 (60.5,67.7)	0.889

**Table 5:** The parental consent rates for HPV vaccination, stratified by status of female students (Bruneian/non-Bruneian) and by district of residence.

Parental Consent rate	Year of Vaccination										p-value
	2012–2015		2012		2013		2014		2015		
	n	% (95% CI)	n	% (95% CI)	n	% (95% CI)	n	% (95% CI)	n	% (95% CI)	
Overall	24257	89.3 (88.9,89.6)	6916	87.5 (86.7,88.2)	6104	89.1 (88.3,89.8)	5715	90.0 (89.2,90.7)	5522	91.0 (90.2,91.7)	0.627
**By status of students**											
Bruneians	24013	94.4 (94.1,94.7)	6872	92.7 (92.1,93.3)	6034	94.6 (94.0,95.2)	5664	94.8 (94.2,95.4)	5443	95.9 (95.3,96.4)	0.662
Non-Bruneians	244	14.0 (12.4,15.7)	44	8.9 (6.6,11.8)	70	14.7 (11.7,18.3)	51	13.6 (10.4,17.5)	79	20.1 (16.3,24.4)	0.336
**By district**											
Brunei-Muara	17515	87.7 (87.2,88.1)	4878	83.9 (83.0,84.9)	4426	87.8 (86.9,88.7)	4209	89.4 (88.5,90.3)	4002	90.6 (89.7,91.5)	0.513
Tutong	2628	97.4 (96.7,97.9)	775	99.0 (97.9,99.5)	665	98.4 (97.0,99.1)	596	95.8 (93.9,97.2)	592	95.8 (93.8,97.2)	0.804
Belait	3412	90.4 (89.4,91.3)	1007	96.3 (94.9,97.3)	854	88.4 (86.2,90.3)	776	88.3 (85.9,90.3)	775	87.9 (85.5,89.9)	0.789
Temburong	702	95.9 (94.1,97.2)	256	95.9 (92.5,97.8)	159	94.6 (89.8,97.4)	134	94.4 (88.8,97.4)	153	98.7 (94.9,99.8)	0.976

## 4 Discussion

In this study, we reported the results of the national school-based HPV vaccination programme for female students of ages 10–17 years. Since the implemention of this programme, 85.8% of all female students in Brunei Darussalam have been fully vaccinated for HPV.

High HPV vaccination coverage rates were also reported across countries that use school-based vaccination programmes, such as in Australia (70%) [9], Bhutan (about 90%), England (83.8%) [20], and Scotland (>80%) [7]. Notably, there is a stark difference in HPV vaccination coverage rates between countries that use school-based vaccination programmes when compared to countries that use community-based vaccination programmes, such as the United States (65.6%) [21] and France (38.9%) [10]. This suggests that school-based approaches are a better and feasible strategy in increasing the HPV vaccination coverage rate at the national level.

We also observed that the vaccination coverage rates increased steadily during the study period. A similar trend was also reported in Canada's school-based vaccination programme [22]. During the first year of school-based HPV vaccination programme, there could have been initial public reservations towards the vaccine as it was recently introduced. However, increased publicity and awareness through talks and activities organised by the Ministry of Health for students and parents would have instilled public confidence on the vaccine. This could explain the higher vaccination coverage rates observed in 2015, when compared to previous years during the study period. Promoting awareness and knowledge, as well as addressing the concerns and misconceptions of parents and children were recommended to increase the vaccination coverage rate [12, 23].

Similarly, we observed higher vaccination coverage rate for the first dose when compared to the second and complete doses. This downward trend is consistent with that reported from school-based programmes in Australia [9] and Canada [8]. On one hand, the high vaccination coverage rate for the fist dose could indicate the wide acceptance of the HPV vaccine [9]. In Brunei, students were given a briefing prior to the administration of first vaccine, which covers the general topics of the vaccine (such as its purpose, importance, and possible side effects). This could give reassurance to students and might have contributed to the high first dose coverage rate. Provider-focused intervention in the form of repeated contact education on HPV vaccination and individualised feedback was reported to further improve the vaccination coverage rates [24]. On the other hand, a considerable decrease in the complete dose coverage rates was mainly due to the issues in reaching students who had missed their vaccine on the scheduled vaccination day at school. In such cases, these students would instead be given appointment to go to school health office to receive their dose. Such inconvenience could have hindered their dose compliance, particularly for those with transport issues (mainly in terms of difficulty on the parents’ side in allocating time or securing transport to the health centre). This was also reported in a similar school-based programmes in Australia [9] and Canada [8]. Another explanation is that some students may have dropped out of school before the end of school year. We have encountered such cases in our programme and given that the main point of contact was at the school-level, this poses similar difficulty issues in reaching them for subsequent doses. There were also few instances where parents decided to discontinue the vaccine, either because their children developed certain adverse reactions towards the vaccine or there were concerned about what they have read on the Internet.

At the district level, we observed that Brunei-Muara had the lowest vaccination coverage rate and parental consent rate. Brunei-Mura is the most populated district in Brunei Darussalam (average of 71.6% of the total population during the study period), has the highest proportion of secondary schools (65.3%) and hence the highest proportion of female students (73.5%). This comparatively high number of eligible students in the Brunei-Muara district could have over-stretched the workforce at School Health Services, such that follow-up visits for those who have missed the vaccination may have been overlooked. In contrast, the manageable number of female students in other districts could be easier to be followed-up. The reasons for the low parental consent rate at Brunei-Muara district are not known, and this warrants a separate investigation into this matter. Mass media campaigns, particularly targeting parents, could be emphasized in this district to increase public awareness on the importance of HPV vaccine.

When stratifying our data into school types, we found a much higher vaccination coverage rates for female students in government schools when compared to private schools. This could be due to the disporportionate number of Bruneian students in government schools (98.0%), when compared to that in private schools (36.7%). Additionally, this observation could be due to the vaccine cost borne by non-Bruneian students. Bruneian students are required to pay a very minimal tuition fee in government schools, while all students are required to pay the full tuition fee in private schools. Also, in both school types, HPV vaccination is provided at no cost to Bruneian students, but at a fee for non-Bruneian students. The fact that non-Bruneian students have to pay for the vaccine may have contributed to low vaccination coverage rates in private schools, as the proportion of non-Bruneian students in private schools are higher than government schools. Furthermore, if they choose to get vaccines from our programme, they have to first visit the district vaccination centre to make the payment. This point could cause certain inconvenience to some parents. Hence, non-Bruneian students may resort to vaccination options other than what was provided by our programme. Also, some parents may opt to vaccinate their children in their home country, as it may be cheaper. Other parents may choose to get their children vaccinated in private health clinics instead, where different vaccine brands were offered. Our programme only offered one vaccine brand for each year, and that the brand usage depends on the acquisition cost for that particular year. These points could also explain the low parental consent rate for non-Bruneian students, when compared to Bruneian students.

The impact of vaccine cost could also be observed in the increase in parental consent rate for non-Bruneian students, whereby parental consent rate increased from 8.9% in 2012 to 20.1% in 2015. From 2014 onwards, the vaccine cost for non-Bruneian students decreased from $200 to $71.95 Brunei dollars for a complete 3-dose vaccination regime. This could be a major factor in the increased of parental consent rate amongst the non-Bruneian students. Indeed, vaccine cost has been previously reported as one barrier of getting HPV vaccine [25, 26]. This decrease in vaccine cost could be a possible explanation for the improved coverage rate during the study period. Another possible explanantion, though likely in a small proportion, are that some may have refused vaccination due to allergy worries. This is because the HPV vaccine contains yeast which may cause anaphylaxis among those with an allergy to yeast [27].

There are a few limitations in this study. Firstly, detailed analysis on the individual-level factors associated with vaccination intake was difficult as the data collected was aggregated at the class-level. Secondly, human error may occur in data collection, as data was manually recorded on paper format. Thirdly, our programme allowed us to track the HPV vaccine uptake status of students who have given or not given consent to join our programme. We are not able to track those who could have obtained their vaccines from private health clinics or overseas.

Although several challenges were encountered during the start of this national vaccination programme, the lessons learnt are invaluable for the subsequent smooth running of the programme. Such challenges were experienced during the actual programme implementation in the field, mainly in terms of logistics and manpower. For the former, we found that it is best to plan the programme at least one year in advance, as this allows time for the procurement of vaccines and equipment (such as pharmaceutical fridges and Automated External Defibrillator), arranging training & schedule of delivery of vaccines, planning of promotional activities, etc. Also, the government and private schools in Brunei have different school term periods. When the programme first started, all schools started their vaccination in January and this affected the administration of the third dose in private schools, as it fell during school holiday period. Therefore, in the subsequent years, the vaccination schedule was adjusted to accommodate the school terms of all schools.

In terms of manpower, there was not enough school health nurses to administer the vaccines in timely manner, particular during the one month gap between first and second dose. Hence help was sought from community- and hospital-based nurses to help deliver the vaccines. In addition, we realised that proper training for the nurses is important. Training that were given to our nurses includes knowledge on the vaccine and cold chain, especially on maintaining cold chain while delivering vaccines to schools. Also, we have encountered high defaulter rates in some schools. In cases where students have difficulty coming to the school health office, we would schedule another date to deliver the vaccine in that particular school.

During the revision of this paper, the school-based vaccination programme is still on-going in Brunei. In this paper, we decided to first report the vaccine coverage results until 2015 due to the change from 3-dose to 2-dose schedule from 2016 onwards. We anticipate that this programme would continue in the near future. To further improve the vaccination coverage rate for complete dose, a survey could be conducted to understand other issues that may have led to lower complete dose coverage rate. Future qualitative studies or surveys on the knowledge and attitudes towards HPV vaccine intake among parents and students would also be useful to determine their perceptions of the vaccine and the reasons for low parental consent rate. Parents’ awarenesss and beliefs about HPV vaccine could influence their decision in givng consent for their daughters to be vaccinated. It was previously reported that factors such as their social demographic background, knowledge regarding HPV and the vaccine may play an important role in their refusal or consent of their daughters to be vaccinated [28].

## 5 Conclusion

In conclusion, the national school-based HPV vaccination programme has achieved an overall complete dose coverage rate of 85.8%. Hence, this is an effective way in delivering HPV vaccination to females in this age range, as part of the cervical cancer prevention and control programme. We have identified two issues that have lowered the overall coverage rate: vaccine cost and diffculty to reach students who have missed their scheduled vaccination in schools. The programme can be further improved by identifying other barriers of accepting and completing their HPV vaccine dose schedule.

## Declaration

### Author contribution statement

Liling Chaw: Conceived and designed the experiments; Analyzed and interpreted the data; Contributed reagents, materials, analysis tools or data; Wrote the paper.

Shaun Lim Tze Wei: Performed the experiments; Analyzed and interpreted the data; Contributed reagents, materials, analysis tools or data; Wrote the paper.

Siti Rosemawati Md Yussof: Conceived and designed the experiments; Performed the experiments; Analyzed and interpreted the data; Wrote the paper.

### Funding statement

This research did not receive any specific grant from funding agencies in the public, commercial, or not-for-profit sectors.

### Competing interest statement

The authors declare no conflict of interest.

### Additional information

No additional information is available for this paper.
